# Intestinal, Airway, and Cardiovascular Relaxant Activities of Thymoquinone

**DOI:** 10.1155/2012/305319

**Published:** 2012-12-18

**Authors:** Muhammad Nabeel Ghayur, Anwarul Hassan Gilani, Luke Jeffrey Janssen

**Affiliations:** ^1^Department of Biological and Biomedical Sciences, Aga Khan University, Sind, Karachi 74800, Pakistan; ^2^Department of Medicine, St. Joseph's Hospital, McMaster University, Room T3338, 50 Charlton Avenue East, Hamilton, ON, Canada L8N 4A6

## Abstract

Thymoquinone (TQ) is a bioactive component found in many medicinal herbs. In this study, we report the smooth and cardiac muscle relaxant activities of this compound. TQ concentration dependently suppressed spontaneously contracting rabbit jejunum while also relaxed high K^+^-(80 mM) induced contractions in jejunum and guinea-pig ileum, indicating activity at voltage-operated Ca^++^ channels (VOCC). Further, TQ displaced Ca^++^ concentration-response curves, obtained in a Ca^++^-free environment, to the right, showing blockade of VOCC. Similar activity was observed with verapamil, a standard VOCC blocker. TQ also exhibited nonadrenergic relaxation of agonist-induced contractions in guinea-pig trachea. When tested in fluo-4-loaded mouse lung slices, TQ inhibited ACh-induced airway narrowing and Ca^++^ signalling in airway smooth muscle cells. In endothelium-intact and endothelium-denuded rat aorta, TQ inhibited high K^+^-induced contractions at significantly lower concentrations than phenylephrine-(PE-) (1 microM) induced contractions. Relaxation of PE-induced contractions was resistant to blockade by L-NAME and atropine. In guinea-pig atria, TQ showed noncholinergic relaxation of atrial force and rate of contractions. These data suggest smooth and cardiac muscle relaxant activity of TQ possibly mediated, in part, via blockade of VOCC. The results also justify the use of TQ containing plants in related health disorders like colic, diarrhoea, cough, and asthma.

## 1. Introduction

**Figure 1:**
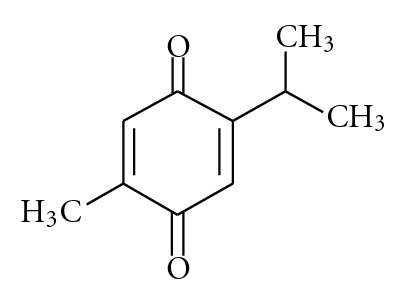
Chemical structure of thymoquinone (C_10_H_12_O_2_; molecular weight 164.20).

Thymoquinone (IUPAC name: 2-Isopropyl-5-methylbenzo-1,4-quinone); TQ is an aromatic ketone (Figure 1) found in many medicinal plants. It is known to be an active phytochemical constituent in seeds of *Nigella sativa* or black cumin [1], whole plant of *Satureja Montana* or savory [2], and in essential oils of *Monarda fistulosa* or wild bergamot [3]. All these plants are known for their traditional therapeutic value in diseases of the gastrointestinal tract and airways. Black cumin is used in colic, cough, asthma, and bronchitis [4]; savory is known for its spasmolytic, antidiarrheal, anticolic, and expectorant potential [4], while wild bergamot is useful in gut disorders, cough, and bronchitis [4]. As TQ is a known constituent of these medicinal plants, it is worthwhile to investigate the pharmacology of this compound.

TQ has been reported for its therapeutic potential in a number of medical conditions. Most notably, it is regarded as a potent antioxidant [5, 6] as well as known for its analgesic and antiinflammatory [7], nephroprotective [8], hepatoprotective [5, 6], neuroprotective [9], and anticancer [10] properties. To further explore the pharmacology of this therapeutically active compound, we tested it on different standard isolated smooth and cardiac muscle preparations. We found that TQ exhibits gut spasmolytic, tracheal, and airway relaxant (looking at Ca^++^ signalling in airway smooth muscle cells, or ASMC, using fluo-4-loaded mouse lung slices), vasodilator and relaxant activities on the cardiac muscles mediated most possibly, at least in part, via blockade of Ca^++^ influx into the cells through voltage-operated Ca^++^ channels (VOCC).

## 2. Methods

### 2.1. Animals

 Experiments performed complied with the rulings of the Institute of Laboratory Animal Resources, Commission on Life Sciences, National Research Council and were approved by the Ethics Review Committee of the Aga Khan University and Research Ethics Boards of McMaster University and St. Joseph's Hospital. Experiments were done using local rabbits (~1 kg; either sex), guinea pigs (500–600 g; either sex), Sprague-Dawley rats (170–200 g; male), and Balb-C mice (6–8 months old; female). These were housed in animal quarters at Aga Khan University (rabbits, guinea pigs, and rats) and St. Joseph's Hospital (mice) in environmentally controlled (23–25°C) and specific pathogen-free conditions. The animals were given a standard chow ad libitum and allowed free access to tap water.

### 2.2. Drugs and Reagents

TQ, the subject of this study, was obtained from the Sigma Chemical Company (St. Louis, MO, USA). The following reference chemicals were obtained from the source specified: acetylcholine chloride (ACh), atropine sulphate, carbamylcholine chloride (carbachol, CCh), isoprenaline hydrochloride, *N*_*ω*_-nitro-L-arginine methyl ester hydrochloride (L-NAME), phenylephrine hydrochloride (PE), propranolol hydrochloride, and verapamil hydrochloride (Sigma Chemical Company). The following chemicals were used to make physiological salt solutions: potassium chloride (Sigma Chemical Company), calcium chloride, glucose, magnesium chloride, magnesium sulphate, potassium dihydrogen phosphate, sodium bicarbonate, sodium chloride, sodium dihydrogen phosphate (E. Merck, Darmstadt, Germany), and ethylene glycol tetraacetic acid (EGTA) (BDH Laboratory Supplies, Poole, England). All chemicals used were of the highest purity grade.

Cell culture reagents were obtained from Invitrogen Life Technologies–GIBCO (Carlsbad, CA, USA). Dulbecco- modified Eagle's medium (DMEM) for slice incubation was supplemented with PennStrep (penicillin 10,000 units/mL, streptomycin 10,000 *μ*g/mL), amphotericin B (125 *μ*g), L-ascorbic acid 35 *μ*g/mL, transferrin 5 *μ*g/mL, selenium 3.25 ng/mL, and insulin 2.85 *μ*g/mL. All reagents were obtained from Sigma-Aldrich (St. Louis, MO, USA) unless specified otherwise. Hanks-balanced salt solution (HBSS; Invitrogen Inc., Burlington, ON, Canada) was supplemented with 4-(2-hydroxyethyl)-1-piperazine ethanesulfonic acid buffer (HEPES, 0.02 M) and was titrated to pH 7.4 with NaOH at 37°C. Agarose-type VII solution (4% w/v, Sigma-Aldrich) was dissolved in distilled water at 60°C, cooled to 37°C, and mixed with 2X HBSS to give a 2% agarose-HBSS solution at 37°C.

### 2.3. Isolated Rabbit Jejunum Preparation

Experiments were performed as described earlier [11]. Segments of rabbit jejunum (2 cm long) were suspended in 10 mL tissue baths containing Tyrode's solution, aerated with a mixture of 95% oxygen and 5% carbon dioxide (carbogen), and maintained at 37°C. The composition of Tyrode's solution in mM was 2.68 KCl, 136.9 NaCl, 1.05 MgCl_2_, 11.90 NaHCO_3_, 0.42 NaH_2_PO_4_, 1.8 CaCl_2_, and 5.55 glucose. Isotonic intestinal responses were recorded using Harvard student oscillographs and force transducers. Each tissue was allowed to equilibrate for at least 30 min before the addition of drugs. Under these conditions, rabbit jejunum exhibits spontaneous rhythmic contractions, allowing testing of relaxant (spasmolytic) activity directly without the use of an agonist. Any potential contractile effect of test material was assessed as percent of maximum effect produced by control drug, ACh (10 *μ*M), while inhibitory effect was measured as percent change in spontaneous contractions of rabbit jejunum obtained immediately before the addition of the test compound.

### 2.4. Determination of Ca^++^ Antagonist Activity in Isolated Rabbit Jejunum

To assess whether the spasmolytic activity of test compound was mediated through Ca^++^ channel blockade, high K^+^ (80 mM) concentration was used to depolarize the rabbit jejunum preparations as described by Farre et al. [12]. High K^+^ was added to the tissue bath, which produced a sustained contraction in the muscle preparation. Test compound was then added to the tissue bath in a cumulative fashion to obtain a concentration-dependent inhibitory response [13]. The relaxation of jejunum preparation precontracted with high K^+^ was expressed as percent of the control response mediated by high K^+^. To possibly confirm this Ca^++^ antagonist activity of the test compound, tissue was allowed to stabilize in normal Tyrode's solution, which was then replaced with Ca^++^-free Tyrode's solution containing EGTA (0.1 mM) for 30 min in order to remove all Ca^++^ from the tissue. This solution was further replaced with a K^+^-rich and Ca^++^-free Tyrode's solution, having the following composition: KCl 50, NaCl 91.04, MgCl_2_ 1.05, NaHCO_3_ 11.90, NaH_2_PO_4_ 0.42, glucose 5.55, and EGTA 0.1 mM. Following an incubation period of 30 min, control concentration-response curves (CRCs) of Ca^++^, added into the tissue bath, were constructed. When the control CRCs of Ca^++^ were found super imposable (usually after two cycles), the tissue was pretreated with the test compound for 60 min to confirm the possible Ca^++^ channel blocking effect. The CRCs of Ca^++^ were then reconstructed in the presence of increasing concentrations of the test compound. Verapamil was used as a positive control.

### 2.5. Isolated Guinea-Pig Ileum Preparation

Guinea-pig ileum segments 2 cm long were mounted in 10 mL tissue baths containing Tyrode's solution, aerated with carbogen, and maintained at 37°C. Isotonic responses were recorded on Harvard student oscillographs. Under these conditions, ileum behaves as a quiescent preparation and helps in evaluation of substances with contractile activity. A preload of 1 g was applied to each tissue and kept constant throughout the experiment. Following an equilibration period of 30 min, isotonic contractions to ACh (0.3 *μ*M) were repeated to stabilize the preparation. An agonist contact time of 20 sec was used, together with a 3 min interval between doses. Sustained contractions, with high K^+^ (80 mM), were also induced to test for possible relaxant activity.

### 2.6. Isolated Guinea-Pig Trachea Preparation

Guinea-pig tracheal tubes were dissected out and kept in Kreb's solution with composition (mM) of NaCl 118.2, NaHCO_3_ 25.0, CaCl_2_ 2.5, KCl 4.7, KH_2_PO_4_ 1.3, MgSO_4_ 1.2, and glucose 11.7; pH 7.4. Tracheal tube was cut into rings, 2-3 mm wide, each containing 2 cartilage rings. Each tracheal ring was opened by a longitudinal cut on the ventral side opposite to the smooth muscle layer, forming a tracheal strip with a central part of smooth muscle in between the cartilaginous portions on the edges [14]. The preparation was then mounted in a 20 mL tissue bath containing Kreb's solution maintained at 37°C and aerated with carbogen gas. A preload tension of 1 g was applied to each of the tracheal strips. The tissue was equilibrated for 1 hr, after which contractile responses to submaximal concentrations of CCh (1 *μ*M), intervals of 45 min, were recorded until reproducible responses were obtained. We then tested the effect of the test compound on resting baseline tension of the tracheal strip as well as against CCh (1 *μ*M) and high K^+^-(80 mM) induced contractions. To test for an involvement of *β*-adrenergic receptors in the relaxant effect of the test compound, the tissues were pretreated for 1 hr with propranolol (1 *μ*M), and response of test compound was repeated in the presence of the antagonist upon CCh-induced contractions.

### 2.7. Airway Contractility and [Ca^++^]_*i*_ in Mouse Lung Slices: Preparation of Slices

Lung slices were prepared as previously described in mice [15, 16]. Mice were euthanized by CO_2_ followed by terminal exsanguination. The trachea was exposed and cannulated using a blunt-ended 19 G needle, followed by chest wall removal to expose the lungs. The lungs were inflated with approximately 1.2 mL agarose (2% in HBSS; 37°C). To clear the airway lumen, 0.2 mL of air was injected to flush the agarose-HBSS solution out of the airways into the alveolar tissue. The lungs were rinsed with 4°C 1X HBSS and the whole mouse was kept at 4°C for 15 minutes. The lungs were then removed and placed in 4°C HBSS for an additional 30 minutes to ensure the complete gelling of the agarose within the lungs. The lungs were separated into individual lobes and bathed in cold HBSS. Slices (approximately 120 *μ*m thick) were cut in 4°C HBSS with an EMS-4000 Tissue Slicer (Electron Microscopy Sciences, Hatfield, PA, USA) from the right upper lobe, transferred to room temperature HBSS until all slices were obtained from the lobe, then transferred to DMEM, and incubated overnight at 37°C.

### 2.8. Airway Contractility and [Ca^++^]_*i*_ in Mouse Lung Slices: Ca^++^ Fluorimetry

Experiments were performed as described previously [16]. Lung slices were selected for study only if: (a) the airway of interest was free of agarose, (b) beating of cilia was observed, and (c) the epithelium of the airway was intact. In each group of experiments, slices from different mice were used. For use in confocal laser-scanning microscope, the slices were loaded for 1 h at 37°C with the Ca^++^-sensitive fluorescent probe, fluo-4 AM (7 *μ*M; Molecular Probes, Eugene, OR, USA) dissolved in dimethyl sulphoxide with 0.01% pluronic F-127 added to enhance solubility [17]. The slices were then mounted between two glass cover slips, held in position by a piece of a nylon mesh (CMN-300-B, Small Parts, Miami Lakes, FL, USA), and placed on the stage of a custom-built confocal microscope equipped with a 20X objective. The bathing solution for all experiments was carbogen-aerated 1X HBSS at 37°C which was exchanged constantly via superfusion throughout the experiment. Tissues were washed with 1X HBSS for 30 min prior to the start of experiment to remove extracellular fluo-4 AM.

### 2.9. Airway Contractility and [Ca^++^]_*i*_ in Mouse Lung Slices: Image Acquisition

The tissues were illuminated using 488 nm light from a 20 mW photodiode laser (Coherent Technologies, Palestine, TX, USA), and two distinct images were collected simultaneously: one comprised the light emitted by the dye (only wavelengths greater than 500 nm, using a long-pass filter) to indicate the changes in intracellular Ca^++^ concentration ([Ca^++^]_*i*_) within individual cells (giving the fluorescent image), as well as the 488 nm transmitted laser light passing through the tissue to provide structural details of the whole tissue (giving the laser image). Images were formed and visualized on a computer screen as emitted (fluorescent) and transmitted (laser) images using the recording software Video Savant (IO Industries Inc., London, ON, Canada). Changes in airway diameter (average diameter range is 145–235 *μ*m) were determined by drawing a straight line across the airway in the transmitted image, and a line scan was performed to obtain change in diameter at that particular position. Exposure of the tissue to the laser light during the experiment was limited to the image collection period using a computer-controlled shutter to minimize photobleaching. Frames were captured in time-lapse at a rate of one frame per second and were stored as TIF stacks of several hundred frames. The digitized images for each time point following stimulation were analyzed by selecting regions of interest of 10 × 10 pixels for measurement of [Ca^++^]_*i*_, while cell pixel intensities were analyzed frame by frame using custom-written macros in the image analysis software Scion (Scion Corporation, Frederick, MD, USA). Fluorescence intensities were saved and plotted against time in SigmaPlot (Systat Software Inc., Point Richmond, CA, USA). An increase in fluorescence intensity was interpreted as an increase in [Ca^++^]_*i*_. Autofluorescence was evaluated in nonloaded preparations and was found to be less than 5% of the fluorescence of fluo-4-loaded tissues.

After the tissue was superfused with HBSS for 30 min, baseline [Ca^++^]_*i*_ images were recorded. Later, ACh (10^−5^ M) was added in the perfusion chamber and superfused over the lung slice preparation for around 2 min to record the effect of ACh on airway diameter and Ca^++^ handling (change in [Ca^++^]_*i*_) in ASMC. The effect of vehicle control, alone, on airway diameter and average fluorescence intensity (AFI) were also determined as was the effect of test substance, alone, on baseline airway diameter and AFI. The slice was then washed and superfused with HBSS, after which it was incubated with TQ for 40 min in order to test for its effect on the ACh-induced change in [Ca^++^]_*i*_ and airway diameter. Later, the ACh response was repeated in presence of TQ. Additionally, to rule out any reduction in the response of ACh due to receptor desensitization, the effect of ACh was repeated, after 30 min, in the presence of only the vehicle. After recording these responses on whole airway, we were able to review the video recordings and focus on single ASMC surrounding the airways which had earlier been shown to respond to ACh: in this way the response of ACh, alone or in the presence of the blockers, could be analyzed at the level of single ASMC.

### 2.10. Isolated Endothelium-Intact and Endothelium-Denuded Rat Aorta Preparation

The procedure of Furchgott and Zawadzki [18] was followed with some modifications. Care was observed in isolating the thoracic rat aorta to avoid any damage to endothelium. Rings, 3 mm wide, were mounted in 5 mL tissue baths with Kreb's solution at 37°C and aerated with carbogen gas. A preload of 1 g was applied to the tissue preparations and were allowed to incubate for 30 min. Changes in tension were recorded via World Precision Instrument's (WPI) isometric force transducers (Fort 100) connected to Transbridge 4M and displayed on to a personal computer via CVMS Data Acquisition System. Following an equilibration period of 30 min, the tissues were stabilized with repeated concentrations of PE (1 *μ*M). After stabilization, an induced contraction was obtained with PE (1 *μ*M). Once a plateau was achieved, ACh (0.1 *μ*M) was administered upon this PE-induced contraction to confirm endothelium-dependent relaxation. The endothelium lining of the tissues was removed by gentle rubbing, which resulted in the disappearance of this relaxation. To study a potential vasodilator effect of the test compound, the following strategies were followed.

1. Determine the vasodilator effect of test compound in endothelium-intact preparations upon PE-(1 *μ*M) and high K^+^-(80 mM) induced contractions.
2. Test the vasodilator effect of compound in L-NAME (0.1 mM; for 60 min) and atropine (1 *μ*M; for 20 min) pretreated PE-(1 *μ*M) contracted endothelium-intact preparations.
3. Determine vasodilator effect of test compound; this time on endothelium-denuded preparations upon PE-(1 *μ*M) induced contractions.

### 2.11. Isolated Guinea-Pig Atria Preparation

Experiments were carried out as previously described [19]. Isolated right and left atria from guinea pigs were mounted separately in 20 mL tissue baths containing Kreb's solution maintained at 32°C (unsteady recording at temperature >32°C) and aerated with carbogen gas. The tissues were allowed to beat spontaneously under the resting tension of 1 g. An equilibrium period of 30 min was given before the application of any drug. Control responses of ACh (0.1–0.3 *μ*M) and isoprenaline (0.1 *μ*M) were obtained at least in duplicate. Tension changes in the tissue were recorded via a Grass force-displacement transducer (model FT-03) using Grass Polygraph (model 7). After looking at a possible response of the pure compound in this tissue, this response would be challenged in the presence of atropine 1 *μ*M (20 min) in order to determine involvement of muscarinic receptors in the mode of action.

### 2.12. Data Analysis

All the data are expressed as mean standard error of mean (SEM, *n* = number of experiment) and the half maximal effective concentration (EC_50_) with 95% confidence intervals (CIs). Statistical comparisons were made using one-way analysis of variance (ANOVA) followed by Tukey's Multiple Comparison test or two-way ANOVA as appropriate, with *P* < 0.05 noted as being statistically significant (GraphPad program, GraphPad, San Diego, CA, USA).

## 3. Results

### 3.1. Effect on Isolated Rabbit Jejunum

**Figure 2:**
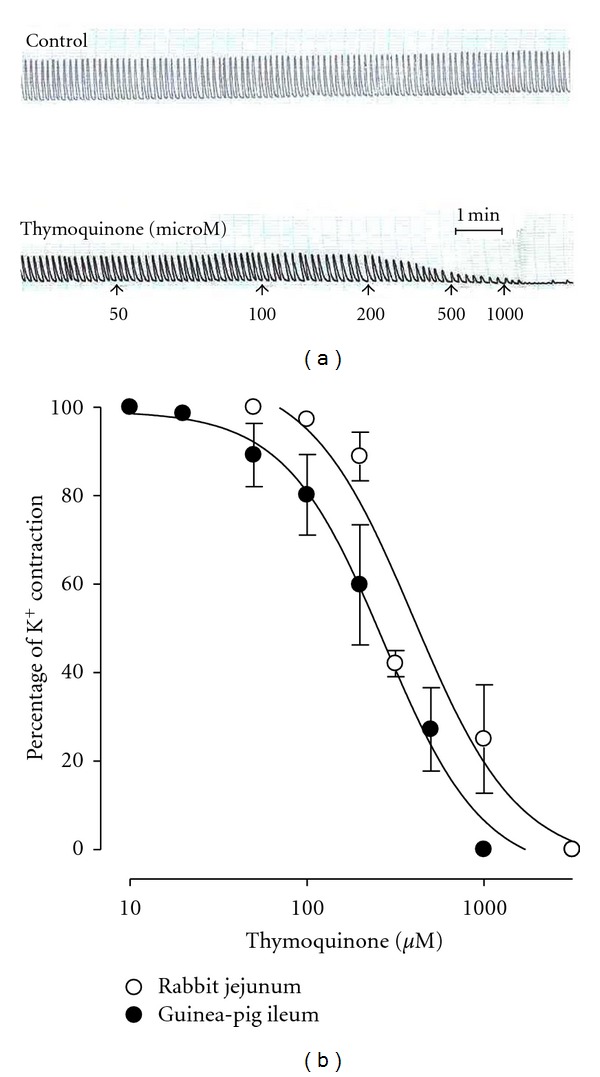
Tracing showing spasmolytic effect of thymoquinone on spontaneously contracting isolated rabbit jejunum preparation (a). (b) shows pooled data for the inhibitory effect of thymoquinone on sustained contractions induced by high K^+^ (80 mM) in isolated rabbit jejunum and guinea-pig ileum preparations. Values shown are mean ± SEM, *n* = 3–5; there is significant difference between both the curves (*P* < 0.001) and between individual concentrations in each curve (*P* < 0.0001), two-way ANOVA.

**Figure 3:**
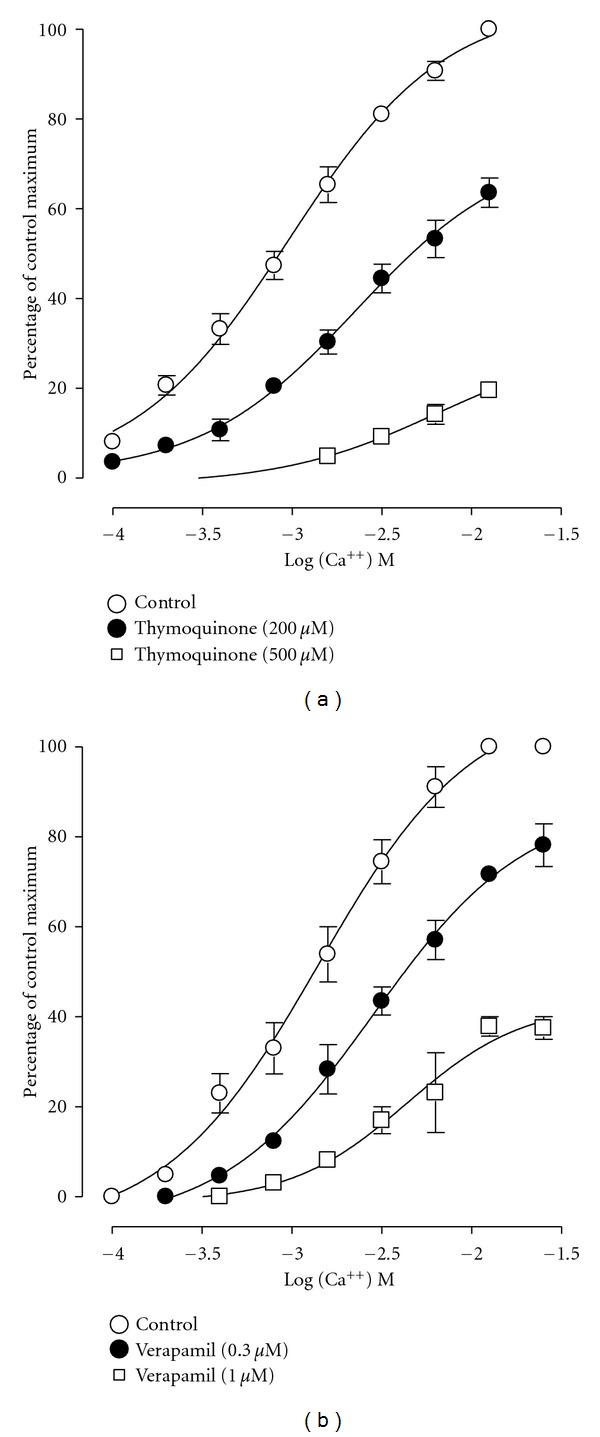
Curves showing the concentration-dependent inhibitory effect of thymoquinone (a) and verapamil (b) on Ca^++^ concentration-response curves, constructed in a Ca^++^-free medium in isolated rabbit jejunum preparations. Values shown are mean ± SEM, *n* = 3-4; there is significant difference between all the curves in (a) and (b) (*P* < 0.0001) and between individual concentrations in all the curves (*P* < 0.0001), two-way ANOVA.

When tested on the spontaneous contractions of rabbit jejunum, TQ was found to be devoid of any stimulant effect and instead caused a concentration-dependent (100–1000 *μ*M) spasmolytic effect (Figure 2(a)), with an EC_50_ of 156.9 *μ*M (95% CI 137.6–178.9; *n* = 5). To further look into this relaxant effect, the response of the compound was tested on high K^+^-(80 mM) induced contractions. TQ exhibited concentration-dependent (100–3000 *μ*M) relaxation of K^+^-induced contractions (Figure 2(b)) with an EC_50_ value of 400.7 *μ*M (95% CI 243.2–659.9; *n* = 5). This potential interaction with Ca^++^ channels was further studied in jejunum. TQ concentration dependently (200–500 *μ*M; *n* = 4) shifted the Ca^++^ CRCs to the right (Figure 3(a)), similar to that produced by verapamil (0.3–1.0 *μ*M; *n* = 3; Figure 3(b)).

### 3.2. Effect on Isolated Guinea-Pig Ileum

TQ tested on the resting baseline tension of guinea-pig ileum did not show any effect up to 1000 *μ*M, thus ruling out the possibility of a stimulant effect of this compound on this tissue preparation. To see if TQ had a relaxant effect, similar to one seen in rabbit jejunum preparation, sustained contractions were induced with high K^+^ (80 mM), allowing acquisition of inhibitory concentration-response data for TQ. The cumulative addition of TQ, to tissue bath, relaxed the high K^+^-induced contractions in guinea-pig ileum (Figure 2(b)) from 50–1000 *μ*M with EC_50_ value of 272.4 *μ*M (95% CI 183.8–403.7; *n* = 3).

### 3.3. Effect on Isolated Guinea-Pig Trachea

**Figure 4:**
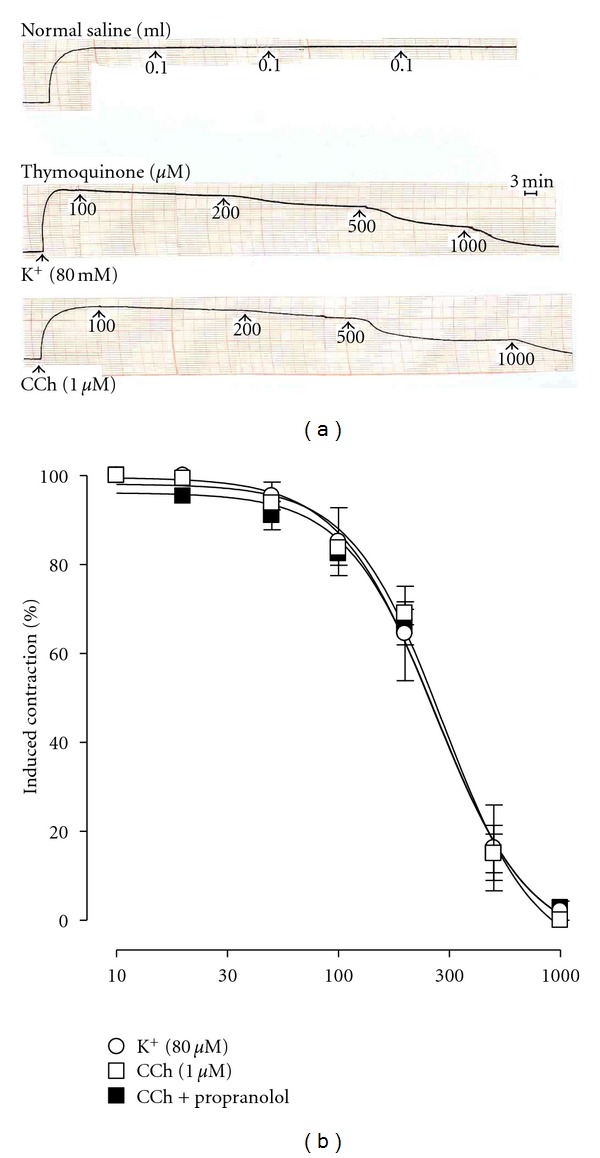
Typical tracing showing the relaxant effect of thymoquinone on high K^+^-(80 mM) and carbachol- (CCh, 1 *μ*M) induced contractions in isolated rabbit trachea preparation (a); upper tracing provides a time control. Pooled data for this relaxant effect of the compound on high K^+^-(80 mM) and carbachol-(CCh, 1 *μ*M) induced contractions is shown in (b). Also shown in (b) is the effect of thymoquinone in the absence and presence of propranolol-(1 *μ*M) on CCh-(1 *μ*M) induced contractions. Values shown are mean ± SEM, *n* = 3–5; there is no difference between the curves (*P* > 0.05), but there is a significant difference between individual concentrations in all the curves (*P* < 0.0001), two-way ANOVA.

TQ was first tested on the resting baseline of the tissue. It was found to be devoid of any effect up to the dose of 1000 *μ*M (not shown; *n* = 3). Later, when tested on high K^+^-(80 mM) and CCh-(1 *μ*M)-induced contractions, TQ concentration dependently (30–1000 *μ*M) inhibited these contractions (Figure 4(a)) with EC_50_ values of 270.1 *μ*M (95% CI 184.0–396.5; *n* = 3) and 295.3 *μ*M (95% CI 254.4–342.7; *n* = 5), respectively, showing no selectivity against any of the agonists (Figure 4(b), *P* > 0.05). This relaxant effect of TQ, when tested on CCh-induced contraction, was found to be resistant to blockade by propranolol (1 *μ*M) (Figure 4(b)).

### 3.4. Mouse Lung Slice Preparation: Effect on Airway Contractility

**Figure 5:**
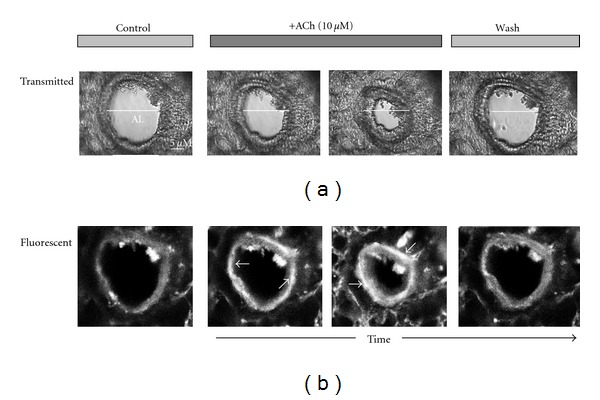
Airway contractile effect of acetylcholine (ACh). Images showing the progressive airway contractile effect of ACh (from left to right), shown in a series of (a) transmitted light (upper panel) and (b) fluorescent (lower panel) images. Note the decrease in the size of airway lumen (AL) in response to ACh administration in the upper panel. White arrows show appearance of bright fluorescent bands (airway smooth muscles) around the airway after ACh administration. Last column shows that the airway diameter and brightness (Ca^++^ levels) return to the baseline after washing the lung slice with physiological salt solution. Time course for the effect of ACh on airway contraction to washout is 2 min.

**Figure 6:**
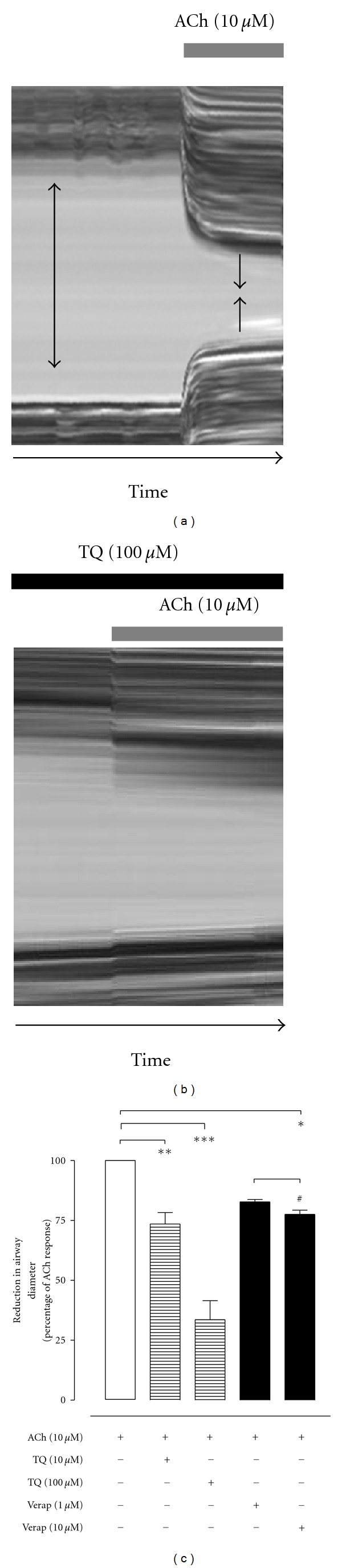
Inhibitory effect of thymoquinone (TQ) on acetylcholine-(ACh, 10 *μ*M) induced reduction in airway diameter in mouse lung slices. (a) and (b) show the effect of ACh, alone (top, a) and in presence (b) of TQ (100 *μ*M), on airway diameter shown as a line scan of a single lung airway. Subfigure (c) shows pooled data for the contractile effect of ACh on airway diameter in the absence (control) and presence of TQ 10 *μ*M (*n* = 6), TQ 100 *μ*M (*n* = 5), verapamil 1 *μ*M (Verap; *n* = 4), and verapamil 10 *μ*M (Verap; *n* = 4). Values shown are mean ± SEM; *P < 0.05, **P < 0.01, ***P < 0.001, and ^#^ *P* > 0.05; one-way ANOVA followed by Tukey's Multiple Comparison test.

ACh (10 *μ*M) produced a marked contraction of mouse airways in the lung slice preparation, seen as reduction in individual airway diameter (Figures 5(a) and 6(a)). The contraction was fast, reproducible, and the airways returned to the baseline diameters once the agonist was washed off. TQ, when given alone (up till 1000 *μ*M) to the lung slice preparations, did not exhibit any effect on resting airway contractility/diameter. When a lung slice was pretreated for 40 min in presence of increasing concentrations of TQ (10–100 *μ*M), a reduction in ACh-induced airway contraction was observed.  Figure 6(b) shows the effect of TQ (100 *μ*M) on ACh-induced airway contraction in one lung slice, while Figure 6(c) shows the pooled data for the effect of 10 and 100 M TQ. The % reduction in airway diameter seen with ACh, in presence of TQ 10 and 100 *μ*M, was 73.5 ± 4.8% (*n* = 6; *P* < 0.01; % of ACh control) and 33.5 ± 8.0% (*n* = 5; *P* < 0.001; % of ACh control), respectively (Figure 6(c)). Verapamil, in concentration of 1 and 10 *μ*M, only marginally attenuated the airway contractile effect produced by ACh (Figure 6(c)). In the presence of verapamil 1 and 10 *μ*M, ACh reduced the airway diameter to 82.7 ± 1.0% (*n* = 4; % of ACh control) and 77.5 ± 1.7% (*n* = 4; *P* < 0.05; % of ACh control), respectively.

### 3.5. Mouse Lung Slice Preparation: Effect on [Ca^++^]_*i*_ in ASMC

**Figure 7:**
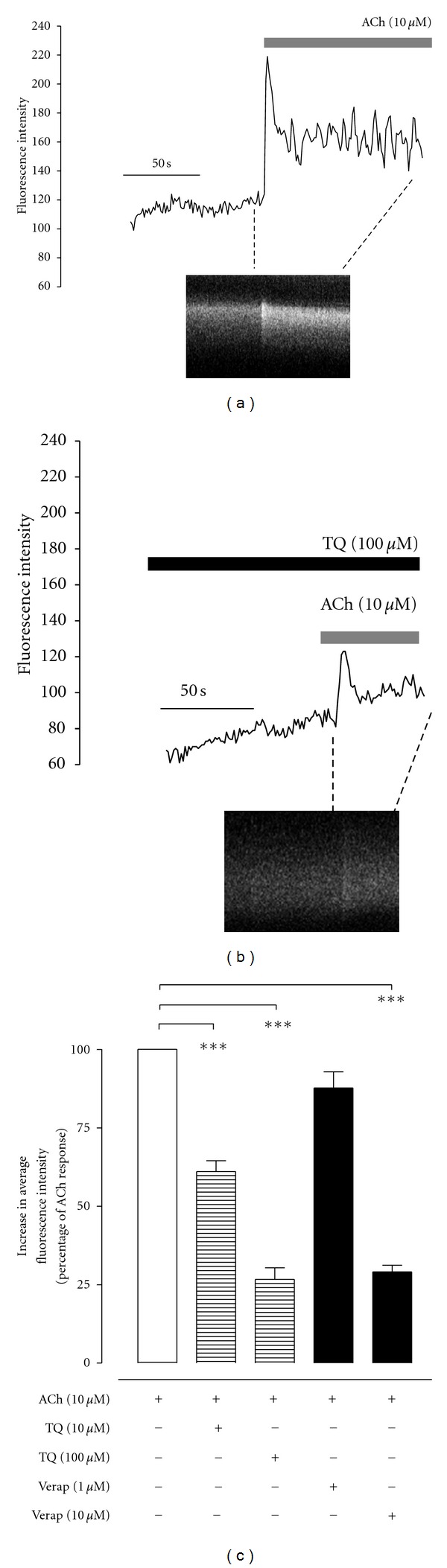
Inhibitory effect of thymoquinone (TQ) on acetylcholine-(ACh, 10 *μ*M) induced elevation of average fluorescence intensity (AFI), representing intracellular Ca^++^ ion concentrations ([Ca^++^]_*i*_), in airway smooth muscle cells (ASMC) studied via fluo-4-loaded mouse lung slices. (a) shows effect of ACh, alone, while (b) shows effect of ACh in presence of TQ (100 *μ*M), on AFI shown as a line graph of the change in AFI and as a line scan of a single ASMC. Subfigure (c) shows pooled data for the effect of ACh on AFI in the absence (control) and presence of TQ (10 *μ*M; *n* = 6), TQ (100 *μ*M; *n* = 5), verapamil (Verap; 1 *μ*M; *n* = 4), and verapamil (Verap; 10 *μ*M; *n* = 4). Values shown are mean ± SEM; ***P < 0.001, one-way ANOVA followed by Tukey's Multiple Comparison test.

In addition to airway contraction, ACh (compared to baseline values) also resulted in elevation of AFI, an indicator of increased [Ca^++^]_*i*_ in ASMC (Figures 5(b) and 7(a)). TQ, in concentrations of 10 and 100 *μ*M, did not affect the baseline AFI but suppressed the ACh-induced Ca^++^ transients and oscillations (shown as reduced fluorescence in line scan of an ASMC and inhibited ACh-induced AFI increase in line graph, Figure 7(b)). The ACh-induced increase in AFI, in the presence 10 and 100 *μ*M TQ, was 61.1 ± 3.4 (*n* = 6, *P* < 0.001, % of ACh response) and 26.7 ± 3.7 (*n* = 5, *P* < 0.001), respectively. Pooled data showing the % increase in fluorescence by ACh alone, and in the presence of increasing concentrations of TQ, is given in Figure 7(c). Verapamil in concentrations of 1 *μ*M and 10 *μ*M, similar to TQ, inhibited ACh-induced elevation of AFI (Figure 7(c)) to 87.7 ± 1.1 (*n* = 4; % of ACh control) and 29.1 ± 2.1 (*n* = 4; *P* < 0.001, % of ACh control), respectively.

### 3.6. Effect on Isolated Rat Aorta

**Figure 8:**
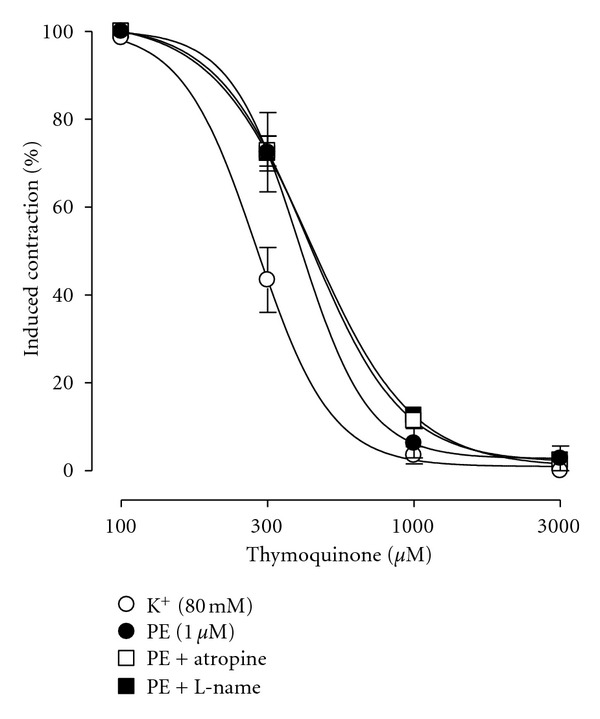
Graph showing concentration-dependent relaxant effect of thymoquinone on high K^+^-(80 mM) and phenylephrine-(PE, 1 *μ*M) induced contractions in isolated endothelium-intact rat aorta. Graph also shows vasodilator effect of thymoquinone in the absence and presence of atropine (1 *μ*M) and L-NAME (0.1 mM) on PE-(1 *μ*M) induced contractions. Values shown are mean ± SEM, *n* = 4-5; there is a significant difference between the two curves with “bold symbols” (*P* < 0.05), while no difference between all the three PE curves (*P* > 0.05). There is a significant difference between individual concentrations in all the curves (*P* < 0.0001), two-way ANOVA.

TQ was devoid of any activity on resting baseline tension of the tissue up to the highest concentration tested (1000 *μ*M) (not shown). When tested against PE-(1 *μ*M) induced contractions, TQ concentration dependently (300–3000 *μ*M) relaxed the agonist-induced contractions (Figure 8) with an EC_50_ value of 406.2 *μ*M (95% CI 310.6–531.2; *n* = 5). The tissue was later pretreated with L-NAME, and TQ was retested. TQ again relaxed the preparation (300–3000 *μ*M) at similar concentrations (Figure 8). There was no significant difference between the concentration-response curves of TQ in the absence or presence of L-NAME (*P* > 0.05). Additionally, neither pretreatment of tissues with atropine (1 *μ*M) (Figure 8), nor denuding the tissues of endothelium (data not shown), had any change on potency or efficacy of vasodilator activity of TQ.

When TQ was tested upon high K^+^-(80 mM) induced contractions, a concentration-dependent relaxation (300–3000 *μ*M) was observed (Figure 8) with an EC_50_ value of 290.1 *μ*M (95% CI 255.3–329.8; *n* = 4). The vasodilator effect of TQ on high K^+^-induced contractions was exhibited at concentrations significantly lower than those for PE-induced contractions (*P* < 0.05; see Figure 8).

### 3.7. Effect on Isolated Guinea-Pig Atria

**Figure 9:**
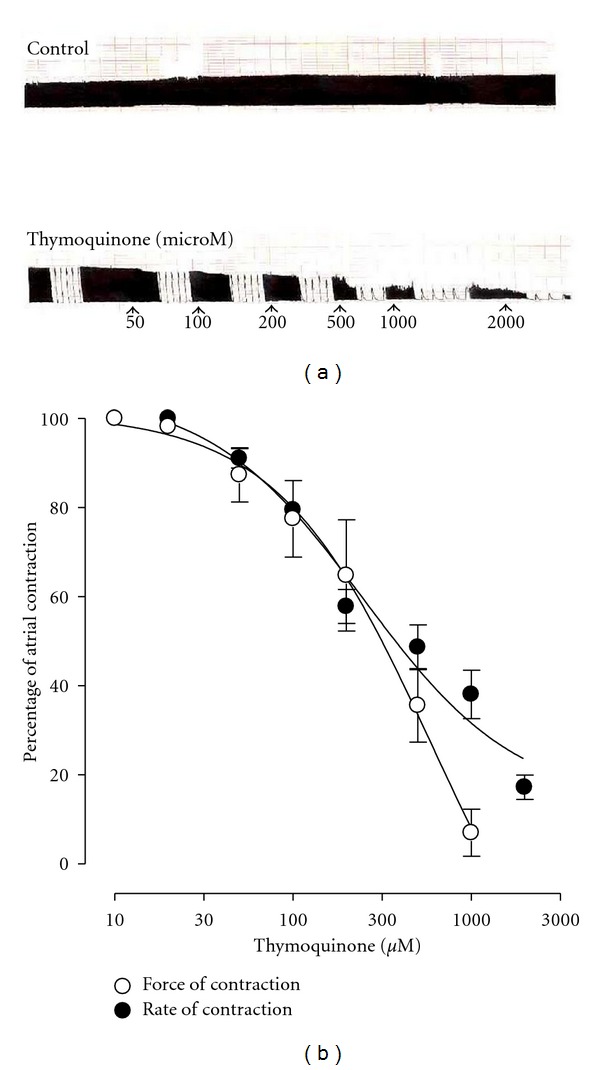
Typical tracing showing the concentration-dependent relaxant effect of thymoquinone on force and rate of spontaneous contractions in isolated guinea-pig atrium (a). Pooled data for this negative inotropic and chronotropic effect of the compound is shown in (b). Values shown are mean ± SEM, *n* = 4-5; there is no difference between the curves (*P* > 0.05), but there is a significant difference between individual concentrations in both the curves (*P* < 0.0001), two-way ANOVA.

TQ exhibited a concentration-dependent relaxation of the force (50–1000 *μ*M) and rate (50–2000 *μ*M) of atrial contractions (Figure 9(a)) with EC_50_ values of 609.1 *μ*M (95% CI 212.1–1749.0; *n* = 5) and 246.4 *μ*M (95% CI 140.6–432.0; *n* = 4), respectively. There was no significant difference (*P* > 0.05) between the relaxant effect of TQ on the force or rate of atrial contractions (Figure 9(b)). The relaxant effect of the compound was resistant to blockade by atropine (1 *μ*M) (data not shown).

## 4. Discussion

TQ showed a concentration-dependent inhibitory effect on the spontaneously beating jejunal preparation indicating intestinal smooth muscle relaxant activity. The spontaneous contractions of smooth muscles, including that of rabbit jejunum, are dependent upon an increase in the cytoplasmic free Ca^++^, which activates the contractile elements [20]. In order to investigate the possible mechanism of this relaxant action, sustained contractions were obtained with high K^+^ (80 mM) which involves entry of Ca^++^ into the cells through VOCC [21]. The compound, when tested on high K^+^-induced contractions, caused a concentration-dependent relaxation possibly indicating restricted entry of Ca^++^ via VOCC, thus showing a Ca^++^ antagonistic effect [20]. To further look into this mechanism, rabbit jejunum tissues and the surrounding media were rendered Ca^++^-free by use of EGTA, a Ca^++^ chelator, and Ca^++^ CRCs were constructed by externally administering Ca^++^ into the tissue baths in the absence or presence of TQ [22]. Under these conditions, externally administered Ca^++^ causes contraction of tissues by entering via VOCC [23]. TQ shifted the Ca^++^ CRCs to the right, further indicating the possible ability of the compound to block entry of Ca^++^ via VOCC. This effect of TQ was similar to verapamil which is a standard intestinal spasmolytic agent with VOCC antagonist effect [24].

TQ was tested on another isolated intestinal preparation that is of guinea-pig ileum. As expected from the results seen in jejunum, the compound showed no contractile effect although ileum is known to be more sensitive to substances with contractile activity [25]. When tested on high K^+^-induced contractions, TQ showed concentration-dependent relaxation, similar to that seen in jejunum. Previously Al-Majed et al. [26] showed that TQ-mediated relaxation of histamine- and serotonin-contracted guinea-pig ileum was through a combination of pathways: (1) inhibition of lipoxygenase products of arachidonic acid metabolism and (2) through an undisclosed nonspecific mechanism. By the help of results obtained in our study in intestinal jejunal and ileal tissues, we can now possibly indicate that the nonspecific mechanism for TQ action could be through blockade of VOCC. For the gastrointestinal tract, TQ has been reported to have gastroprotective activity particularly in colitis [27] and gastric mucosal injury [28]. Our results showing spasmolytic activity of TQ might also explain the beneficial effects of TQ-containing medicinal plants such as black cumin, savory and wild bergamot in hyperactive states of gut.

For determining the activity of TQ in respiratory tissues, it was initially tested on isolated guinea-pig trachea and then later in fluorescence dye-loaded mouse lung slices. While TQ was devoid of any contractile effect on baseline tone in the isolated tracheal preparation, its concentration-dependently suppressed different agonist-induced contractions. TQ relaxed the induced contractions of high K^+^ and CCh at similar potency. The tracheal relaxant effect was resistant to blockade by propranolol, a nonspecific *β*-adrenergic blocker [29] thus ruling out such a mechanism in its effect.

To further look into the airway relaxant effect of TQ and its relationship with Ca^++^ signalling, we screened the compound on fluorescence-dye-(fluo-4-) loaded mouse lung slices and were able to focus on the effect of TQ on Ca^++^ handling and whole airway contraction in ASMC. ACh produced an increase in AFI which consisted of a sharp rise in [Ca^++^]_*i*_ (Ca^++^ transient, needed to establish the airway tension at time of airway contraction), followed by a drop in this level and then a sustained plateau with multiple Ca^++^ oscillations (needed to maintain the tension during airway contraction). These ACh-induced Ca^++^ transients and oscillations, in ASMC have been reported in the literature previously [15, 30]. TQ, in increasing concentrations, inhibited both ACh-induced Ca^++^ transients and oscillations thus indicating that it might be interfering with Ca^++^ signalling in airways. In murine ASMC, an ACh-induced increase in [Ca^++^]_*i*_ is known to be caused: (1) indirectly through influx of extracellular Ca^++^ through Ca^++^ channels on plasma membrane and (2) directly through release of Ca^++^ from the intracellular Ca^++^ stores [15]. This means that TQ is possibly interfering with either/both of these mechanisms to inhibit the ACh-induced increase in [Ca^++^]_*i*_. Verapamil, a standard VOCC antagonist, also inhibited the ACh-induced increase in [Ca^++^]_*i*_ possibly via its ability to stop Ca^++^ influx into the cells and thus halt the refilling of internal Ca^++^ stores. VOCC antagonists also abolish high K^+^-induced increase in [Ca^++^]_*i*_ in mouse ASMC [30]. On airway contraction, ACh showed a profound contractile effect. This contractile effect of ACh was inhibited by increasing concentrations of TQ but, interestingly, not by verapamil. Unlike TQ, verapamil only marginally inhibited the ACh-induced airway contraction, and there was no significant difference between the effects of two increasing concentrations of verapamil on the ACh-induced airway contraction (*P* > 0.05). Thus, TQ showed a similar effect to verapamil on ACh-induced increase in Ca^++^ transients and oscillations but not the cholinergic mechanical response. This shows a difference in the modes of action of TQ and verapamil in the mouse AMSC. Perez and Sanderson [30] have shown that high concentrations of K^+^, which enhance inward movement of Ca^++^ through opening of VOCC in mouse ASMC, elicit changes in AFI but hardly show any significant change in mouse airway constriction. This could be a reason as to why in our results, verapamil did inhibit the ACh-induced increase in AFI but not the ACh-induced airway contraction. This also reiterates the fact why blockers of VOCC are hardly used clinically in asthma. On the contrary, TQ showed an inhibitory effect, unlike that of verapamil that was able to significantly inhibit Ca^++^ transients and oscillation and airway contraction. This airway relaxant effect of TQ also explains the traditional use of its parent medicinal plants like black cumin, savory and wild bergamot in hyperactive states of respiratory system like cough and asthma. Some previous studies [26, 31] have shown a tracheal relaxant effect of TQ. Without specifying, these studies had indicated presence of an additional mechanism. We have successfully shown that TQ not only interferes with Ca^++^ signalling pathways but also inhibits airway contraction. TQ has also been shown to have an antiinflammatory action [32, 33] and therapeutic effect in animal models of asthma [34, 35]. The tracheal and airway relaxant effect of TQ in this study, together with its reported antiinflammatory actions, indicates its benefit in asthma therapy. Asthma is a chronic disease characterized by inflammation and hypersensitivity of airway smooth muscles to different spasmogens [36].

TQ was also tested on cardiovascular preparations of rat aorta and guinea-pig atria. Rat aorta was used to examine the effect of TQ on vascular resistance. Rat aorta is a standard tissue used for determining endothelium-dependent and -independent vasodilation [37]. TQ was found devoid of any vasoconstrictor effect on vascular baseline tone, but when tested on the endothelium-intact rat aorta precontracted with PE or high K^+^, it inhibited sustained contractions induced by both the agonists indicating that TQ is blocking both the Ca^++^ influx pathways. TQ was more potent in relaxing high K^+^ than PE-induced contractions, indicating preference for VOCC than receptor-operated Ca^++^ channels (ROCC) [38]. PE induces Ca^++^ influx via ROCC, while high K^+^ induces Ca^++^ influx via VOCC [39]. The vascular endothelium plays a pivotal role in modulating the contractility of vascular smooth muscle through the release of vasodilator and constrictor factors [40]. One such vasodilator factor is nitric oxide (NO), while the muscarinic M_3_ receptors are one set of receptors through the activation of which NO is released [18]. To observe any possible nitrergic and/or muscarinic involvement in the vasodilator effect of TQ, tissues were separately preincubated with L-NAME, a standard nitric oxide synthase inhibitor [41], and atropine, a muscarinic receptor blocker [42]. This resulted in no change in the vasodilator effect of TQ indicating absence of such mechanisms in TQ's vasodilator effect. An endothelium-independent involvement in TQ's vasodilator effect was further confirmed when denuding the aorta preparation of endothelium also did not make any difference in the vascular relaxant effect of TQ (data not shown). Thus, this points to the presence of possibly only a Ca^++^ antagonistic mode of effect as evident from the ability of TQ to relax the high K^+^-induced contractions at lower concentrations, compared to PE. A similar vasorelaxant effect of TQ, but in isolated rat pulmonary arterial rings, was recently reported by Suddek [43] who also showed a nonnitrergic/nonmuscarinic relaxant effect of TQ. Suddek [43] concluded that TQ exhibits a nonspecific vasorelaxant effect as evident by its inhibitory effect against contractions induced by multiple agonists. We have shown here that the nonspecific relaxant quality in TQ might involve blockade of VOCC.

Lastly we examined the effect of TQ on cardiac contractility using guinea-pig atrial preparations. TQ relaxed the force and rate of spontaneous atrial contractions in a concentration-dependent manner. This inhibitory effect on cardiac tissues was resistant to blockade by atropine indicating an action independent of muscarinic receptor activation. We could not investigate the mechanism of this effect any further. Our observation of a possible cardiovascular relaxant effect of TQ adds to its already reported cardiovascular therapeutic effects such as in hypertension and renal damage [44], cyclophosphamide-induced cardiotoxicity [45], methionine-induced hyperhomocysteinemia [46], and atherosclerosis [47].

In summary, our results show spasmolytic activity of TQ in isolated smooth muscle preparations of gastrointestinal, airway, and vascular smooth muscle tissues, while also in cardiac muscle preparations. While these results are novel and add new information to the already available pharmacological data on this compound, they also help to justify the use of TQ containing medicinal plants like black cumin, savory and wild bergamot in muscular hyperactive disorders of gastrointestinal, and airway and cardiovascular systems. More detailed studies are in progress to further investigate the mechanism of muscle relaxant activity of TQ.
